# Effect of Implant Surface Roughness and Macro- and Micro-Structural Composition on Wear and Metal Particles Released

**DOI:** 10.3390/ma14226800

**Published:** 2021-11-11

**Authors:** Andrea El Hassanin, Giuseppe Quaremba, Pasquale Sammartino, Daniela Adamo, Alessandra Miniello, Gaetano Marenzi

**Affiliations:** 1 Department of Chemical, Materials and Industrial Production Engineering, University of Naples “Federico II”, P.le Tecchio 80, 80125 Naples, Italy; andreahelassanin@unina.it; 2 Department of Industrial Engineering, University of Naples “Federico II”, Via Claudio 21, 80125 Naples, Italy; quaremba@unina.it; 3 School of Specialization in Oral Surgery, University of Campania “L. Vanvitelli”, Via L. De Crecchio 6, 80100 Naples, Italy; pasqualesammartino91@gmail.com; 4 Department of Neurosciences, Reproductive and Odontostomatological Sciences, University of Naples “Federico II”, Via Pansini 5, 80131 Naples, Italy; daniela.adamo@unina.it; 5 School of Specialization in Oral Surgery, University of Naples “Federico II”, Via Pansini 5, 80131 Naples, Italy; miniello.alessandra@libero.it

**Keywords:** implant surface, roughness, osteointegration

## Abstract

Background: Considerations about implant surface wear and metal particles released during implant placement have been reported. However, little is known about implant surface macro- and microstructural components, which can influence these events. The aim of this research was to investigate accurately the surface morphology and chemical composition of commercially available dental implants, by means of multivariate and multidimensional statistical analysis, in order to predict their effect on wear onset and particle release during implant placement. Methods: The implant surface characterization (roughness, texture) was carried out through Confocal Microscopy and SEM-EDS analysis; the quantitative surface quality variables (amplitude and hybrid roughness parameters) were statistically analyzed through post hoc Bonferroni’s test for pair comparisons. Results: The parameters used by discriminant analysis evidenced several differences in terms of implant surface roughness between the examined fixtures. In relation to the observed surface quality, some of the investigated implants showed the presence of residuals due to the industrial surface treatments. Conclusions: Many structural components of the dental implant surface can influence the wear onset and particles released during the implant placement.

## 1. Introduction

Dental implants are commonly used in daily practice for functional and aesthetic rehabilitation after tooth loss [1]. Titanium and its alloy are the most commonly used materials due to their biocompatibility, mechanical characteristics and chemical stability [2]. However, many studies do not focus on the properties of dental implant materials when they are broken down to smaller particle [3,4,5]. As a consequence of implant placement, microfractures and compression can occur at the bone side and the implant surface can be simultaneously subjected to a combination of torsional and frictional forces, which may alter the original implant surface. [5,6]. Stress concentration on the implant surface can destroy the titanium oxide layer on the implant and wares the cover favoring the release of titanium particles in the surrounding tissues [7]. However, little is known about macro and microstructural components of the dental implant surface which can influence the wear onset and the particles released during implant placement. In order to increase the contact area between the living bone and the fixture, manufacturers have introduced many surface treatments, namely chemical (acid-etching) and mechanical (sandblasting) or a combination of the two [8,9,10,11,12,13,14,15]. Rough titanium implant surfaces can also be obtained through material addition, using technologies such as Thermal Spray Processing (usually Titanium Plasma Spray, TPS), sometimes mixed with the previously mentioned techniques [16]. Among the available chemical methods, etching has become widely accepted and is based on the controlled corrosion of the implant surface through strong acids (hydrofluoric, nitric, sulfuric) [17]. This has been reported to produce micro-holes (dimples) with 1.5–2 µm in size on the implant surface, which assist osteointegration by increasing the available surface area for the attachment of bone tissue [17,18]. Mechanical grinding based methods mainly refers to sandblasting in this scenario, which involves the projection of abrasive ceramic particles such as alumina, titanium dioxide and calcium phosphate, ranging in size between 25 and 75 µm, through a suitable carrier fluid, which is typically air [19]. The TPS technique also ensures a very rough surface (macro-roughness of up to 240 µm and the micro-roughness approximately 40 µm), based on the overlapping droplets of solidified titanium [16]. These different manufacturing techniques promote different surface topographies at both the micro- and nano-scales and superficial chemical compositions. At present, none of the previous industrial techniques proposed to increase surface roughness is able to prevent the release of titanium particles from the implant surface following insertion into the bone [19]. Suarez-Lopez Del Amo et al. evidenced how all system showed small angular or round elongated titanium debris especially in the crestal part of the osteotomy site [19]. Deppe et al. reported how during implant placement, the surfaces obtained with subtractive modifications appeared to suffer less wear and particle loosening than surfaces with additive modification [20,21]. Wennerberg et al. found that moderately rough surface presented more titanium release from implant surface than smooth surface their size decreased with the increase of distance from the titanium implant [22]. Generally, these particles are highly difficult to eliminate from the peri-implant tissues and their concentration can be able to procure local inflammation and modify the osteoblast and osteoclast balance [7]. These particles, in fact, have been proved to inhibit the differentiation of osteoblast precursor cells and promote the bone resorption function of the osteoclast by inducing the differentiation of osteoclast [7]. They may also be transported away from the bone-implant interface causing inflammation in distant tissues, with potential systemic involvement [5]. Pathological alterations of the peri-implant tissue could also be caused by factor other than the implant material itself. A titanium surface can be contaminated by various substances used in the manufacturing process, such as cutting fluid, sandblasting powder or etching agents [23,24]. The aim of this research was to investigate accurately the surface morphology and chemical composition of commercially available dental implants, in order to predict their effect on wear onset and particle release during implant placement.

## 2. Materials and Methods

**Figure 1:**
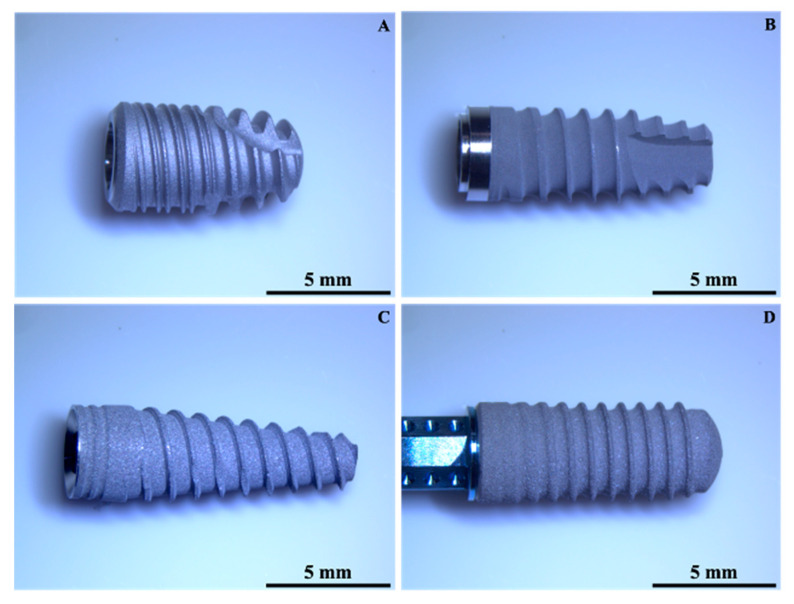
Low magnification images of the investigated dental implants: (**A**) = Global D; (**B**) = Sweden & Martina; (**C**) = Globalwin; (**D**) = Straumann (Magnification 6.7×).

All the examined implants had a tronco-conical shape; only the In-Kone implant (Global D, France; for short, A) was made of titanium Grade 4 (CPTi), whereas the Premium fixture (Sweden & Martina, Artigianale Cornegliana, Italy; for short, B) and Globalwin implant (Biosafin, Trezzano Rosa, Italy; for short, C) were of titanium Grade 5 (Ti6Al4V), and the Roxolid SLActive fixture (Straumann, Basel, Switzerland; for short, D) was made of titanium zirconium alloy (Ti-Zr alloy). Fixtures B, C, and D had a sandblasted/etched surface treatment, while implant A had a sandblasted/double-etched surface treatment. Table 1 reports the main information of the investigated implants and Figure 1 illustrates their related low magnification images (6.7×), obtained with a Nikon SMZ745T stereomicroscope (Nikon, Tokyo, Japan). For the experimental analysis, three implants for each manufacturer were considered. Table 2 reports the mechanical properties of titanium Grade 4 (CPTi) and titanium Grade 5 (Ti6Al4V) [9].

This comparative study evaluated implant surfaces with different characterization (roughness and texture, surface morphology and chemical composition). The quantitative surface description (roughness, texture) was analyzed through a Leica DCM3D Confocal Microscope (Leica Microsystems, Wetzlar, Germany), equipped with the surface processing software LeicaMap v7^®^ (Leica Microsystems, Wetzlar, Germany). In first instance, the 3D surfaces of the implants were acquired at low magnification (10×), along their longitudinal axes. The latter were expressed as colour-coded 3D plots and subsequently elaborated with LeicaMap v7^®^ to perform an analysis of the thread geometry and step of each implant considered by extracting profiles along the implants axes and using the distance measuring function. Subsequently, higher magnification acquisitions (50×) were performed on the same implants to evaluate the surface roughness, considering an area of 500 × 500 µm^2^ taken between two threads. For each implant, six primary profiles were extracted perpendicularly to the implant longitudinal axes within the acquired area, having an evaluation length of 500 µm. The subsequent roughness profiles, obtained after the application of a Gaussian filter with a 0.08 mm cut-off, were used to evaluate the following parameters according to the ISO 4287 standard [25]: mean arithmetic height (Ra), maximum peak-to-valley distance (Rz), maximum peak height (Rp), maximum valley depth (Rv), profile skewness (Rsk) and profile kurtosis (Rku). It is worth to highlight that the first four parameters are descriptive of the height amplitude of the topological events (peaks and valleys) whereas the Rsk and Rku, referred as hybrid parameters, are representative of the symmetry of the peaks and valleys distribution and their tailness, respectively [9,26].

The implants surface analysis was carried out also using a Hitachi TM3000 SEM (Hitachi, Tokyo, Japan) equipped with a 15kV electron beam and an Oxford Instruments Swift ED 3000 EDS probe (Oxford Instruments, Abingdon, UK), in order to compare the quantitative results obtained from confocal microscopy with a qualitative evaluation of the surface morphology deriving from the different surface treatments applied to the selected implants. To this aim, images at 1500× magnification were taken in the same areas investigated through confocal microscopy and EDS analyses were carried out in order to highlight any effect of the surface treatments on the superficial chemical composition of the implants.

The quantitative variables obtained from confocal microscopy (i.e., the amplitude parameters Ra, Rz, Rp, Rv, and the hybrid parameters Rsk, and Rku) were statistically described in terms of mean, standard deviation (SD), and 95% Confidence Interval (CI). The two-way analysis of variance (ANOVA) test to compare the values under investigation was performed. To test the multiple comparisons of the difference of means, Bonferroni’s post hoc test was used.

In order to model the dependent categorical variable (implant manufacturer) based on its relationship to one or more predictors, the Discriminant Analysis (for short, hereafter, DA) was used. Given a set of independent variables, the DA attempts to find linear combinations of those variables that best separate the groups of cases. DA was performed by entering all variables and by selecting, through a “stepwise” method, the best set of discriminating variables. The criterion for controlling the stepwise selection was the maximum Wilks’ lambda defined as:

λ = (Variance Between groups)/(Variance Within group).

This test takes into consideration the differences between all the centroids and the cohesion (homogeneity) within the groups. A maximal solution would require testing every possible subset to determine which would produce the very best results.

The mathematical objective of DA is to weight and linearly combine the discriminating variables in some fashion so that the four groups of manufacturers were forced to be as statistically distinct as possible [27]. The statistical theory of DA assumes that the discriminating variables have a multivariate normal distribution and that they have equal variance-covariance matrices within each group. In practice, the technique is very robust and these assumptions need not be strongly adhered to. The discriminant scores were derived by maximizing the quadratic distance of Mahalanobis from the centroid of the two clusters [28]. The *p*-value level for significance was 0.05, all *p* values are two-sided. Statistical analysis was performed with the software IBM SPSS Statistics, v.20.0 (IBM Corp. Armonk, NY, USA).

## 3. Results

### 3.1. Confocal Microscopy

**Figure 2:**
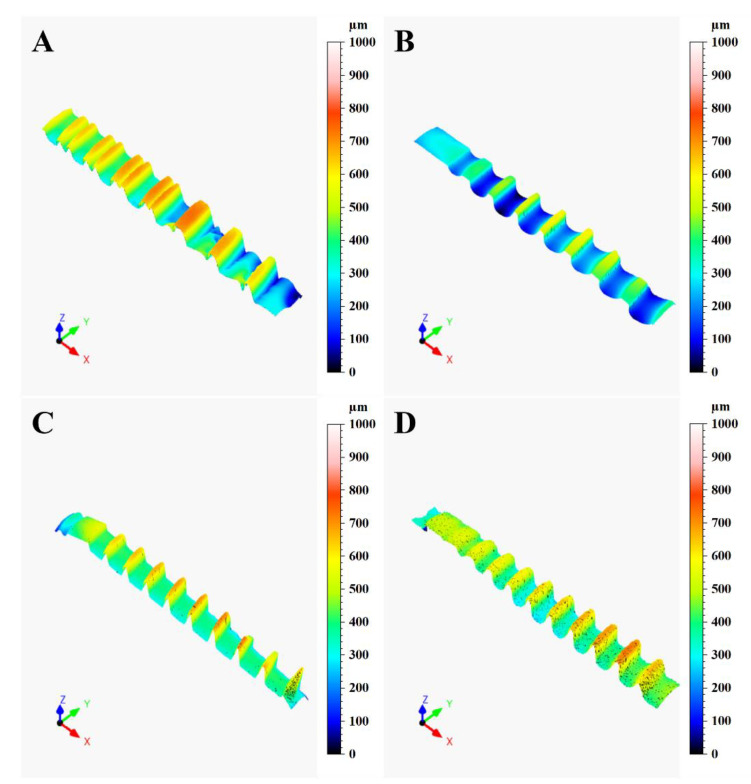
Surface texture comparison of the dental implants acquired with a Leica DCM3D microscope (Leica Microsystems, Wetzlar, Germany): (**A**) = Global D; (**B**) = Sweden & Martina; (**C**) = Globalwin; (**D**) = Straumann (10× magnification).

**Figure 3:**
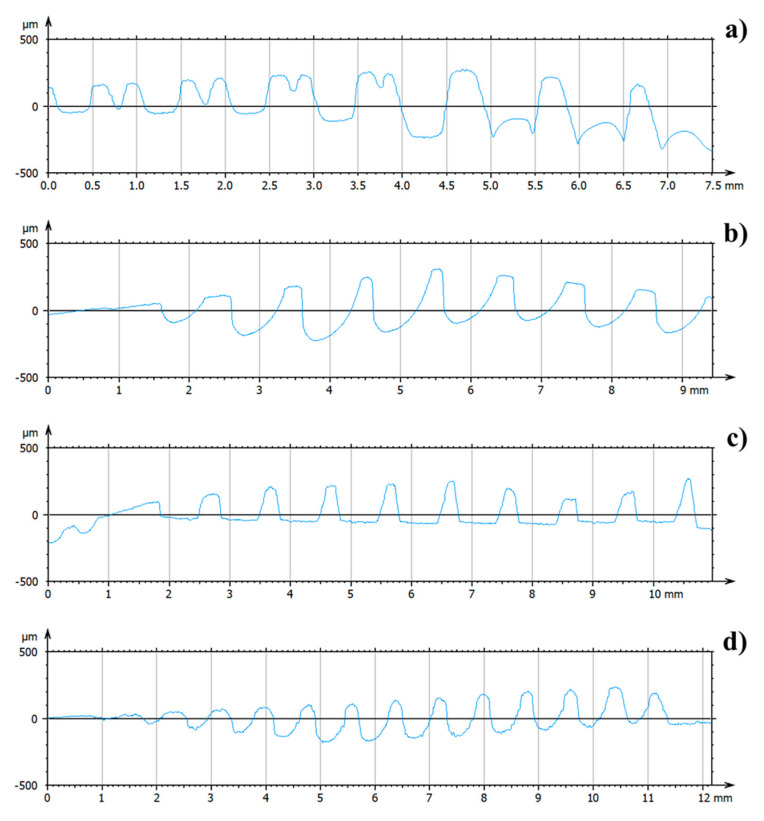
Texture profiles of the implant surface extrapolated using LeicaMap^®^ (Leica Microsystems, Wetzlar, Germany): (**a**) = Global D; (**b**) = Sweden & Martina; (**c**) = Globalwin; (**d**) = Straumann.

Figure 2 shows the 3D colour-coded surface plots of the different implants, used to analyze the implant texture features. From the plots, it is evident that the implants are quite different between each other in different aspects, the first notable one being the presence of a double threaded profile in the A implant and the different thread geometries between the implants (also visible in Figure 1). According to the procedure described in the previous section, a single profile along the implants axes was extracted through the LeicaMap v7 ^®^ software for each implant. Their comparison is illustrated in Figure 3. The results showed the thread pitch of 1 mm for A, B and C fixtures; only the D fixtures presented a thread pitch of 0.8mm. Moreover, the thread geometries were different, being triangular for the C and D implants, square for the B implant and hybrid (double square + single trapezoidal) for the A implant.

Concerning roughness, the results of ANOVA (Table 3) and Bonferroni’s multiple comparisons (Table 4) show a significant difference between the B implant which, in particular, presented the lowest significant mean of roughness (Ra = 0.550, Rz = 3.450, Rp = 1.756, and Rv = 1.656, *p* < 0.001) and D implant representing its highest counterpart (Ra = 2.139, Rz = 11.856, Rp = 6.044, and Rv = 5.811, *p* < 0.001).

**Figure 4:**
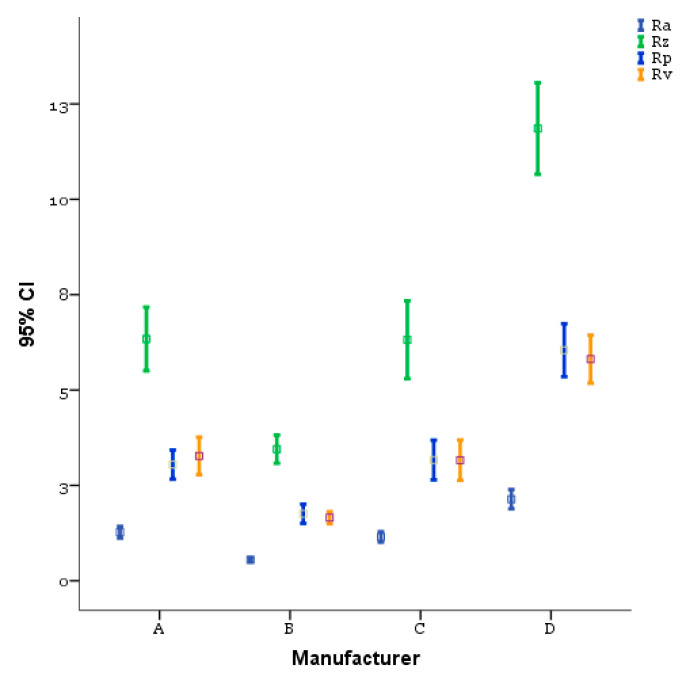
Amplitude parameter comparison for the different dental implants investigated (bars represent 95% CIs, squares represent means).

**Figure 5:**
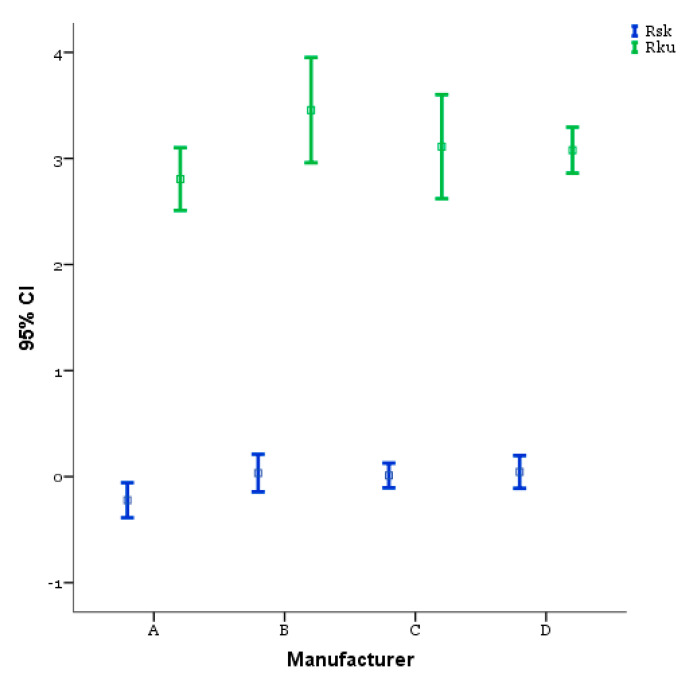
Hybrid parameters comparison for the different dental implants investigated (bars represent 95% CIs, squares represent means).

Figure 4 and Figure 5 illustrate the comparison of the amplitude parameters (Ra, Rp, Rv, Rz) and the hybrid parameters (Rsk, Rku), respectively, for the different implants, obtained from the 50× magnification acquisitions performed with confocal microscopy according to the description provided in the previous section. Concerning roughness, the results of ANOVA (Table 3) and Bonferroni’s multiple comparisons test (Table 4) show a significant difference between the B implant which, in particular, presented the lowest significant mean of roughness (Ra = 0.550, Rz = 3.450, Rp = 1.756, and Rv = 1.656, *p* < 0.001) and D implant representing its highest counterpart (Ra = 2.139, Rz = 11.856, Rp = 6.044, and Rv = 5.811, *p* < 0.001).

On the other hand, in relation to the investigation conditions used in this work, the dental implants produced by A and C (Table 4) led to a comparable surface quality (*p* = 1.00). The hybrid parameters are reported in Figure 5. The ANOVA (Table 5) and Bonferroni’s multiple comparisons test (Table 6) show a good symmetry of the profiles, as supported also from the values of Rsk reported in Figure 5, which were always close to 0 and leading therefore to unperceivable, not significant differences between the implants from this point of view (*p* > 0.05).

In terms of profile kurtosis, the results reported in Figure 5 show that the B implant had the highest value (Rku = 3.456) among the selected implants, despite it represented the lowest roughness case, whereas the other implants had comparable values which were also higher than 3. According to the significance of the Rku parameter [25] and within the case study, the results suggested that the surface treatments of the different dental implants led to the formation of sharp peaks. Moreover, the sharpness of the peaks is a roughness scale-independent factor, justifying the results obtained for the B surface in terms of high Rku and low Ra.

The stepwise procedure begins by selecting the single best-discriminating variable (i.e., Ra) according to determined criterion (i.e., maximize Wilks’ lambda). A second discriminating variable (i.e., Rz) is selected as the variable best able to improve the value of the discrimination criterion in combination with the first variable. The third variable is similarly selected according to its ability to contribute to further discrimination. At each step, variables already selected may be removed if they are found to reduce discrimination when combined with more recently selected variables.

The changes in Wilks’ lambda and their associated chi-square tests of statistical significance as the information in successive discriminant functions is removed are shown in Table 7. This indicates that considerable discriminating power exists in the variables being used (the larger lambda is, the larger discriminant power is present). A very large significant lambda was found in the first and in the second function. This indicates that it would not be useful to derive the third and last discriminant function, since it would not significantly add to the ability to discriminate between the groups. Consequently, the remaining computations were based on only the first two functions.

Nearly all of the variance explained by the model is due to the first two discriminant functions, as shown in Table 8.

The classification results are reported in Table 9; the overall classification rate is quite high, meaning that 72% of selected original grouped cases are correctly classified.

**Figure 6:**
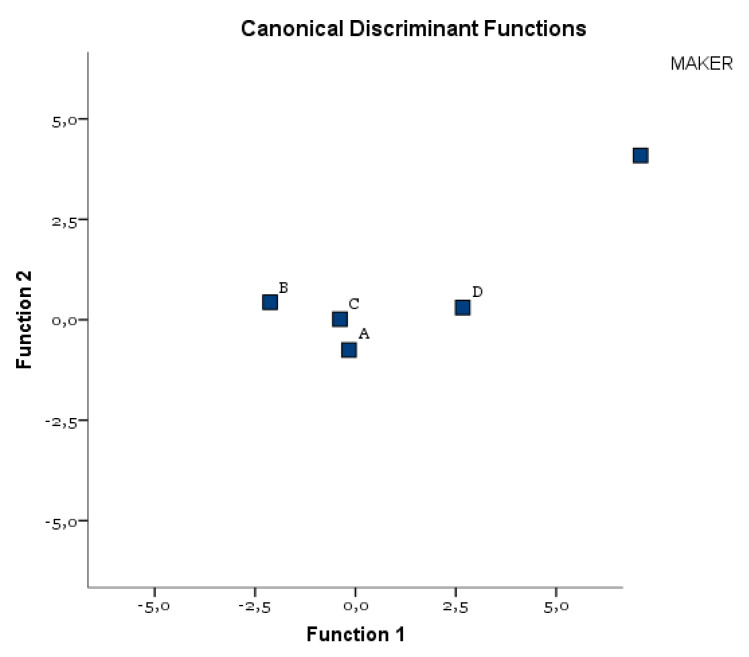
Centroids distribution of the four manufacturers.

The DA identified a binomial (Ra and Rz), whose orthogonal combination allowed us to correctly classify 61% of A, 100% of B, 44% of C, and 83% of D (Table 9). Both C and A implants showed a lower homogeneity of the Ra and Rz parameters. D had, on average, the highest values, B had, on the other hand, the lowest values. Figure 6 shows the all-groups scatter plot.

### 3.2. SEM-EDS

**Figure 7:**
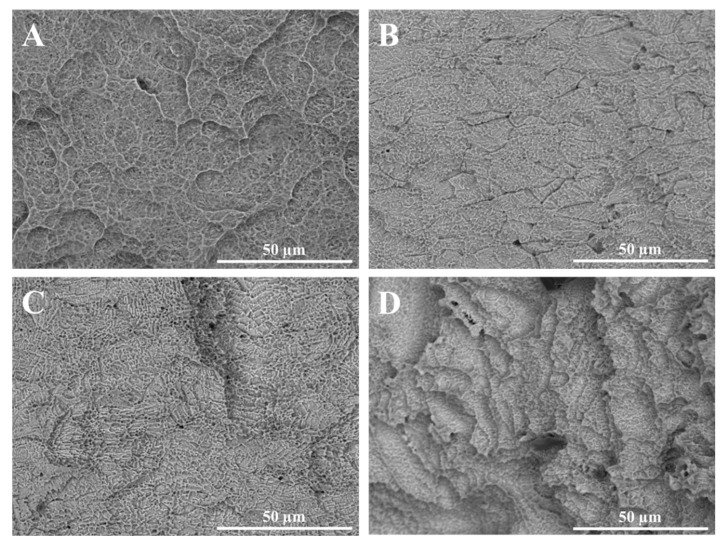
SEM images of the investigated dental implants (1500× magnification) (**A**) Global D; (**B**) Sweden & Martina; (**C**) Globalwin; (**D**) Straumann.

The quantitative results obtained from confocal microscopy were corroborated by the qualitative morphology analysis carried out with SEM-EDS application. According to the SEM images reported in Figure 7, it is conceivable that the same conclusions drawn from the quantitative analysis could also be drawn in this case concerning the surface quality comparison between the implants. More specifically, the differences in the effect of the surface treatments on the surface morphology are clearly visible and they describe well the different surface roughness measured for the selected implants. For instance, A implant presented deeper dimples on the surface due to the double etching after sandblasting whereas the D implant had the most jagged surface. Moreover, the B implant showed the flattest surface with also the smallest dimples. However, the A and C implants presented a different surface morphology, despite the quite similar roughness values: this result could be due to the different sandblasting conditions effects on the two implants, as supported for instance by the presence of deeper craters on the surface of the C implant, which were not observed on A.

**Figure 8:**
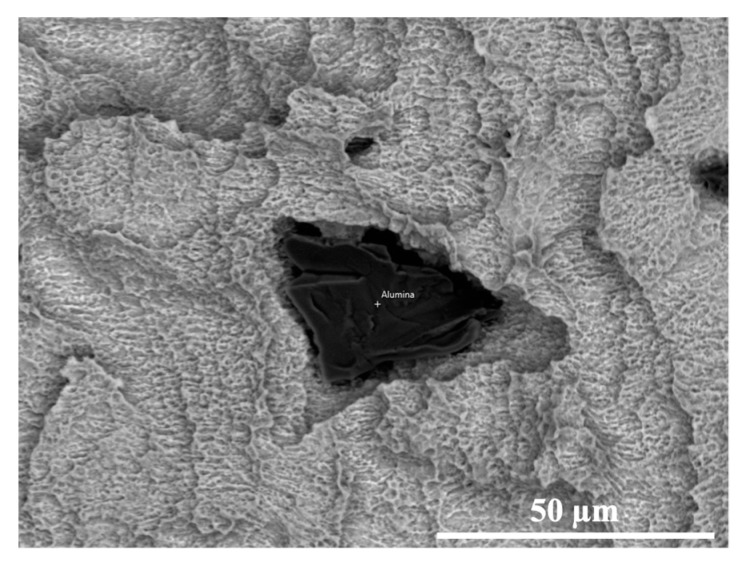
Detailed SEM observation of the D implant surface, showing the presence of entrained alumina particles after the sandblasting treatment (1500× magnification).

In terms of superficial chemical composition, the SEM-EDS analysis proved that all the implants presented only the characteristic elements of the titanium alloys used from the different manufacturers, as reported in Table 6. However, this conclusion was not applicable to the D implant, for which the presence of alumina particles was detected, as illustrated in Figure 8. The latter result, also supported by the EDS results reported in Table 10 given the high aluminium and oxygen wt%, suggests that the subsequent etching step was not able to completely remove the abrasive particles related to the sandblasting step.

## 4. Discussion

This research investigated the surface morphology and chemical composition of commercially available dental implants in order to predict their effect on wear phenomena and titanium particles release during the implant insertion.

Many studies have evidenced how the implant wear, during the implant placement, can be related the surface chemical composition and the extension of the bone–implant contact area [29,30,31].

The Grade 4 titanium (CP Ti) (of the fixture A) had a low wear resistance compared to the Grade 5 titanium (of the implants B and C), which had better mechanical properties that prevented deformation during insertion, reducing the number of surface defects.

Implant A showed double threads for increasing the contact area with the bone and reducing the cortical occlusal stress. This macro-morphology appeared to be more favorable in terms of surface changes during the implant placement than the single-threaded ones, even if it was able to ensure a faster implant insertion [32,33,34,35].

The screw pitch was considered another parameter able to influence the surface area. Previous studies showed that implant surface area increased as screw pitch decreased, in this condition leading to an extension of the bone–implant interface. [36,37]. Kong et al. considered 0.8 mm as the optimal thread pitch for achieving primary stability and optimum stress production [38]. The results show similar thread pitch dimensions between the examined fixtures (1 mm for A, B, and C implants and 0.8 mm for the D implant).

In relation to the observed textures, the investigated implants showed a different thread shape, which was considered another important aspect able to influence the bone–implant contact surface. The square threads (such as those of the C and D implants), assuring the highest contact with the peri-implant bone, can be considered more susceptible to wear onset and the release of metal particles than the other thread designs (triangular shape, such as those of C and D implants) during the implant placement.

The reported results evidence how the different industrial surface processing conditions of titanium surfaces were able to influence the roughness and the chemical composition of the examined implant surfaces.

Based on the results, the chemical treatment seemed to develop the coarsest surface with a rough and sharp-cornered morphology, whilst a wavy morphology occurred with sandblasting (Figure 4). The examined implants were treated with both etching and sandblasting surface treatments. B and C implant surfaces showed less roughness than A and D. Moreover, the latter presented the highest roughness, ensuring a larger contact area between the implant surface and the surrounding bone than the other evaluated implants [9]. Rougher implants with higher peaks were related to an increased number of particles released at the bone–implant interface following insertion into bone [19]. The biological/clinical value of the presence of metal friction residues on peri-implant bone surface is not clear, although it was considered the major factor responsible for aseptic implant loosening [39,40]. In vitro model systems showed that released titanium particles can activate the inflammatory response, resulting in an increased secretion of IL-1B, IL-6, and TNF-α in cultured human macrophages, which induce osteoclastogenesis and inhibit osteblastogenesis and consequently lead to bone resorption [41,42,43]. Additionally, Petterson et al. showed that titanium particles generate a pro-inflammatory response in macrophages, activating the cascade NLPR3 inflammasone caspade-1 and the release of mature IL-1ß [23]. Their cytotoxic effect varied considerably in relation to the dose, size, and geometry [44,45]. Nanoparticles were described as more biologically reactive and more potentially harmful than microparticles because of their greater surface-to-volume ratio [23]. It was suggested that specific ions and proteins coated the TiO2 nanoparticles, favoring their internalization by osteoblasts. Once inside the cell, nanoparticles are able to produce DNA damage and oxidative stress [23]. Choi et al. reported how the osteoblast adhesion, proliferation, and viability were reduced by particles of dimensions < 15 μm. Particles with major dimensions were able to increase the expression of receptor activator of nuclear factor kB ligand (RANKL) and the proteolytic activities of matrix metalloproteinases (MMP) 2 and 9 [46]. The potential biological effect of the metal friction residues is also related to their chemical composition and, consequently, to the chemistry of the implant surface modified by the surface treatments [47,48,49,50]. Based on the results, fixtures A and D had the advantage of assuring a higher bone–implant interface, given that peaks can be more easily damaged during the implant insertion and thus more vulnerable to wear and particle release. Implants with these characteristics require a precise placement without excessive pressure against the osteotomy site [19]. This condition could also be influenced by the manufacturer’s precision (sharpness of the cutting tool), surface deformation, and roughness shown by the surgical drills. Elias et al. showed that surgical drills allowed for easier and smoother insertion of the implant into the high-density bone; in this clinical condition, the implant surface suffered less damage than in the bone types III–IV [51].

With the sandblasting process, performed with particles of different chemical composition, size, and geometry, the implant surface may retain some of the blasting particles. In most of the investigated implants, produced by the combination of etching and sandblasting processes (A, B, C), no residues were identified; this is probably related to the ability of the etching process to eliminate the contaminations procured by the sandblasting treatments. On the contrary, implant D showed a surface contamination with the presence of alumina particles (Figure 8), the latter being residues from the sandblasting finishing step. Its diffusion in the peri-implant bone surface may produce biological perturbations such as the inhibition of bone mineralization, the activation of osteoclast-like cells, and the enhancement of the bone erosion [52,53]. Bertoldi et al. evidenced the association between the Al wear particles with oxidative and inflammatory reactions, including iron-mediated oxidation [54]. Other authors reported the diffusion of Al ions in the peri-implant bone as a potential risk factor for the development of neurological disorders, including Alzheimer’s disease and metabolic bone disease such as osteomalacia [55]. Aluminium is neurotoxic and, in addition to genetic factors, plays a role in the development of Alzheimer’s disease by the contribution to the formation of the characteristic beta-amyloid and neurofibrillary tangles. Thus, a common denominator between Alzheimer’s disease and bone fragility may be a chronic low-grade aluminium intoxication [56].

Several limitations in the current study should be taken into account and the results need to be interpreted with caution: 1. The foreign body reaction triggered by Ti particles is a complex host immune response process involved in various immune cell interactions; their role was only outlined in this study. 2. The small sample size of the examined implants.

The next step in our research will be the assessment of the functionality of the examined implant surfaces by in vitro experiments with osteoblast cell cultures and possibly by in vivo experiments in animal models.

## 5. Conclusions

This work examined and compared the surface characteristics of different commercially available dental implants, with a focus on chemical composition and roughness parameters, in order to predict their effect on wear and metal particle release during implant placement.

Based on the experimental outcomes, the following conclusions can be drawn:

- Many implant’s surface macro-components (thread design, screw pitch) and mi-cro-components (chemical surface composition, roughness, industrial surface treatment) can influence the surface wear during the implant placement.
- The surface wear and metal particle release during the implant placement can be also influenced by the manufacturer’s precision (sharpness of the cutting tool), the bone density, and implant macromorphology.
- The biological/clinical value of the presence of metal friction residues on the pe-ri-implant bone surface is not clear, although it was considered the major factor responsible for aseptic implant loosening.

## Figures and Tables

**Table 1:** Dental implants selected for surface texture investigations.

Implant	Material	Surface Treatment	Length (mm)	Diameter (mm)
A	CP Ti	Sandblasting + double etching	8.5	4.5
B	Ti 6Al4V	Sandblasting + etching	8.5	3.8
C	Ti 6Al4V	Sandblasting + etching	11.5	3.75
D	Ti-Zr alloy	Sandblasting + etching	12	3.3

**Table 2:** Mechanical properties of CPTi and Ti6A14V.

Material	UTS (MPa)	YS0.2 (MPa)	E (GPa)	EL (%)
CpTi	660	590	105	20
Ti6A14V	950	880	113.8	14

**Table 3:** ANOVA results (amplitude parameters).

		Sum of Squares	df	Mean Square	F	Sig.
**Ra**	Between Groups	23.176	3	7.725	68.678	0.000
	Within Groups	7.649	68	0.112		
	Total	30.824	71			
**Rz**	Between Groups	667.486	3	222.495	66.342	0.000
	Within Groups	228.057	68	3.354		
	Total	895.543	71			
**Rp**	Between Groups	177.046	3	59.015	60.571	0.000
	Within Groups	66.253	68	0.974		
	Total	243.299	71			
**Rv**	Between Groups	160.334	3	53.445	55.551	0.000
	Within Groups	65.421	68	0.962		
	Total	225.755	71			

**Table 4:** Results of Bonferroni’s post hoc test (amplitude parameters).

R_a_ Mean (SD)	A 1.272 (0.3121)	B 0.550 (0.1098)	C 1.150 (0.2792)	D 2.139 (0.5124)
**A** Mean difference, *p* value	=	0.7222, <0.001	0.1222, 1.00	−0.8667, <0.001
**B** Mean difference, *p* value	=	=	−0.6000, <0.001	−1.5889, <0.001
**C** Mean difference, *p* value	=	=	=	−0.9889, <0.001
**R** **_z_** **Mean (SD)**	**A** **6.339 (1.6807)**	**B** **3.450 (0.7422)**	**C** **6.317 (2.4120)**	**D** **11.856 (2.4120)**
**A** Mean difference, *p* value	=	2.8889, <0.001	0.0222, 1.00	−5.5167, <0.001
**B** Mean difference, *p* value	=	=	−2.8667, <0.001	−8.4056, <0.001
**C** Mean difference, *p* value	=	=	=	−5.5389, <0.001
**R_p_** **Mean (SD)**	**A** **3.044 (0.7702)**	**B** **1.756 (0.5055)**	**C** **3.167 (1.0460)**	**D** **6.044 (1.3980)**
**A** Mean difference, *p* value	=	1.2889, 0.001	−0.1222, 1.00	−3.0000, <0.001
**B** Mean difference, *p* value	=	=	−1.4111, <0.001	−4.2889, <0.001
**C** Mean difference, *p* value	=	=	=	−2.8778, <0.001
**R_v_** **Mean (SD)**	**A** **3.272 (0.9940)**	**B** **1.656 (0.3203)**	**C** **3.161 (1.0689)**	**D** **5.811 (1.2709)**
**A** Mean difference, *p* value	=	1.6167, <0.001	0.1111, 1.000	−2.5389, <0.001
**B** Mean difference, *p* value	=	=	−1.5056, <0.001	−4.1556, <0.001
**C** Mean difference, *p* value	=	=	=	−2.6500, <0.001

**Table 5:** ANOVA results (hybrid parameters).

		Sum of Squares	df	Mean Square	F	Sig.
**Rsk**	Between Groups	0.867	3	0.289	2.970	0.038
	Within Groups	6.613	68	0.097		
	Total	7.480	71			
**Rku**	Between Groups	3.836	3	1.279	2.035	0.117
	Within Groups	42.723	68	0.628		
	Total	46.559	71			

**Table 6:** Results of Bonferroni’s post hoc test (hybrid parameters).

R_sk_ Mean (SD)	A −0.222 (0.3318)	B 0.033 (0.3565)	C 0.011 (0.2349)	D 0.044 (0.3110)
**A** Mean difference, *p* value	=	−0.2556, 0.099	−0.2333, 0.168	−0.2667, 0.075
**B** Mean difference, *p* value	=	=	0.0222, 1.000	−0.0111, 1.000
**C** Mean difference, *p* value	=	=	=	−0.0333, 1.000
**R_ku_** **Mean (SD)**	**A** **2.806 (0.5955)**	**B** **3.456 (0.9972)**	**C** **3.111 (0.9869)**	**D** **3.078 (0.4360)**
**A** Mean difference, *p* value	=	−0.6500, 0.099	−0.3056, 1.000	−0.2722, 1.000
**B** Mean difference, *p* value	=	=	0.3444, 1.000	0.3778, 0.944
**C** Mean difference, *p* value	=	=	=	0.0333, 1.000

**Table 7:** Discriminating power of discriminant functions for Wilks’ lambda.

Test of Function(s)	Wilks’ Lambda	Chi-Square	df	Sig.
1 through 2	0.198	110.283	6	0.000
2	0.816	13.794	2	0.001

**Table 8:** Eigenvalues and percent of variance explained by the first two models.

Eigenvalues			
Function	Eigenvalue	% of Variance	Cumulative %
1	3.133	93.3	93.3
2	0.225	6.7	100.0

**Table 9:** Classification results of the DA.

Classification Results ^a^							
		Manufacturer	Predicted Group Membership				Total
			A	B	C	D	
Original	Count	A	11	2	4	1	18
		B	0	18	0	0	18
		C	5	3	8	2	18
		D	2	0	1	15	18
	%	A	61.1	11.1	22.2	5.6	100.0
		B	0.0	100.0	0.0	0.0	100.0
		C	27.8	16.7	44.4	11.1	100.0
		D	11.1	0.0	5.6	83.3	100.0

^a.^ 72.2% of original grouped cases correctly classified.

**Table 10:** Chemical composition of dental implant surface.

Element (wt%)	A	B	C	D (Surface)	D (Particle)
Titanium	72.9	90.0	76.9	79.3	3.5
Aluminium	8.7	--	--	--	44.7
Vanadium	3.9	--	--	--	--
Oxygen	14.5	10.0	23.1	8.7	51.4
Zirconium	--	--	--	12.0	0.6

## Data Availability

Data sharing is not applicable for this article.

## References

[1] Matos G.R.M. (2021). Surface roughness of dental implant and osseointegration. J. Maxillofac. Oral. Surg..

[2] Marenzi G., Spagnuolo G., Sammartino J.C., Gasparro R., Rebaudi A., Salerno M. (2019). Micro-scale surface pattering of titanium dental implants by anodization in the presence of modifying salts. Materials.

[3] Velasco-Ortega E., Ortiz-García I., Jiménez-Guerra A., Monsalve-Guil L., Munoz-Guzon F., Perez R.A., Gil F.J. (2019). Comparison between sandblasted acid-etched and oxidized titanium dental implants: In vivo study. Int. J. Mol. Sci..

[4] Tuan R.S., Lee F.Y.-I., Konttinen Y.T., Wilkinson J.M., Smith R.L. (2008). What are the local and systemic biologic reactions and mediators to wear debris, and what host factors determine or modulate the biologic response to wear particles?. J. Am. Acad. Orthop. Surg..

[5] Romanos G.E., Fischer G.A., Delgado-Ruiz R. (2019). Titanium wear of dental implants from placement, under loading and maintenance protocols. Int. J. Mol. Sci..

[6] Delgado-Ruiz R., Romanos G. (2018). Potential causes of titanium particle and ion release in implant dentistry: A systematic review. Int. J. Mol. Sci..

[7] Zhou Z., Shi Q., Wang J., Chen X., Hao Y., Zhang Y., Wang X. (2021). The unfavorable role of titanium particles released from dental implants. Nanotheranostics.

[8] Silva G.A.F., Faot F., da Silva W.J., Del Bel Cury A.A. (2021). Does implant surface hydrophilicity influence the maintenance of surface integrity after insertion into low-density artificial bone?. Dent. Mater..

[9] Marenzi G., Impero F., Scherillo F., Sammartino J.C., Squillace A., Spagnuolo G. (2019). Effect of different surface treatments on titanium dental implant micro-morphology. Materials.

[10] Iorio-Siciliano V., Marenzi G., Blasi A., Mignogna J., Cafiero C., Wang H.L., Sammartino G. (2016). Influence of platform-switched, laser-microtextured implant on marginal bone level: A 24-month case series study. Int. J. Oral Maxillofac. Implants.

[11] Cafiero C., Marenzi G., Blasi A., Iorio Siciliano V., Nicolò M., Sammartino G. (2013). Soft and hard tissues healing at immediate transmucosal implants placed into molar extraction sites with collagen membrane uncovered: A 12-month prospective study. Implant Dent..

[12] Lee J., Lee J.B., Yun J., Rhyu I.C., Lee Y.M., Lee S.M., Lee M.K., Kim B., Kim P., Koo K.T. (2021). The impact of surface treatment in 3-dimensional printed implants for early osseointegration: A comparison study of three different surfaces. Sci. Rep..

[13] Zhang W.J., Liu Y., Sun X.Y., Zhao O., Quan H.X. (2021). Clinical effect evaluation of anodized and sandblasted, large-grit, acid-etched implants within 10 years: A meta-analysis. Zhonghua Kou Qiang Yi Xue Za Zhi..

[14] Liu Z., Liu X., Ramakrishna S. (2021). Surface engineering of biomaterials in orthopedic and dental implants: Strategies to improve osteointegration, bacteriostatic and bactericidal activities. Biotechnol. J..

[15] Ullah I., Siddiqui M.A., Liu H., Kolawole S.K., Zhang J., Zhang S., Ren L., Yang K. (2020). Mechanical, biological, and antibacterial characteristics of plasma-sprayed (Sr, Zn) substituted hydroxyapatite coating. ACS Biomater. Sci. Eng..

[16] Pimenta J., Szmukler-Moncler S., Raigrodski A.J. (2021). Physical characterization of 3 implant systems made of distinct materials with distinct surfaces. J. Prosthet. Dent..

[17] Mata A., Su X., Fleischman A.J., Roy S., Banks B.A., Miller S.K., Midura R.J. (2003). Osteoblast attachment to a textured surface in the absence of exogenous adhesion proteins. IEEE Trans. Nanobiosci..

[18] Rüger M., Gensior T.J., Herren C., Von Walter M., Ocklenburg C., Marx R., Erli H.J. (2010). The removal of Al2O3 particles from grit-blasted titanium implant surfaces: Effects on biocompatibility, osseointegration and interface strength in vivo. Acta Biomater..

[19] Suarez-Lopez Del Amo F., Garaicoa-Pazmino C., Fretwurst T. (2018). Dental Implants-associated release of titanium particles: A systematic review. Clin. Oral Implants Res..

[20] Deppe H., Grunberg C., Thomas M., Sculean A., Benner K.U., Bauer F.J.M. (2015). Surface morphology analysis of dental implants following insertion into bone using scanning electron microscopy: A pilot study. Clin. Oral Implants Res..

[21] Marenzi G., Sammartino J.C., Quaremba G., Graziano V., Hassanin A.E., Qorri M.E., Sammartino G., Iorio-Siciliano V. (2018). Clinical influence of micromorphological structure of dental implant bone drills. Biomed. Res. Int..

[22] Wennerberg A., Ide-Ektessabi A., Hatkamata S., Sawase T., Johansson C., Albrektsson T., Martinelli A., Sodervall U., Odelius H. (2004). Titanium release from implants prepared with different surface roughness. An in vitro and in vivo study. Clin. Oral Implants Res..

[23] Mombelli A., Hashim D., Cionca N. (2018). What is the impact of titanium particles and biocorrosion on implant survival and complications? A critical review. Clin. Oral Implants Res..

[24] Bonsignore L.A., Goldberg V.M., Greenfield E.M. (2015). Machine oil inhibits the osseointegration of orthopaedic implants by impairing osteoblast attachment and spreading. J. Orthop. Res..

[25] (1998). ISO4287, BSI Standards Publication Geometrical Product Specifications (GPS)—Surface Texture: Profile Method: Terms, Definitions and Surface Texture Parameters.

[26] Thomas T.R. (1981). Characterization of surface roughness. Precis. Eng..

[27] Tatsuoka M.M., Timm N.H. (2004). Multivariate analysis. Applied Multivariate Analysis. Discriminant and Classification Analysis.

[28] Tinsley H., Brown S. (2000). Handbook of Applied Multivariate, Statistics and Mathematical Modeling.

[29] Niinomi M. (2008). Mechanical biocompatibilities of titanium alloys for biomedical applications. J. Mech. Behav. Biomed. Mater..

[30] Elias C.N., Fernandes D.J., de Souza F.M., Montiero E., De Biasi R.S. (2019). Machanica and clinical properties of titanium and titanium based alloy (Ti G2, TiG4 cold worked nanostrustructured and Ti G5) for biomedical applications. J. Mater. Res. Technol..

[31] Silva G.A.F., Faot F., Possebon A.P.D.R., da Silva W.J., Del Bel Cury A.A. (2021). Effect of macrogeometry and bone type on insertion torque, primary stability, surface topography damage and titanium release of dental implants during surgical insertion into artificial bone. J. Mech. Behav. Biomed. Mater..

[32] Abuhussein H., Pagni G., Rebaudi A., Wang H.-L. (2010). The effect of thread pattern upon implant osseointegration. Clin. Oral Implants Res..

[33] Menini M., Bagnasco F., Calimodio I., Di Tullio N., Delucchi F., Baldi D., Pera F. (2020). Influence of implant thread morphology on primary stability: A prospective clinical study. Biomed. Res. Int..

[34] Mattheos N., Li X., Zampelis A., Ma L., Janda M. (2016). Investigating the micromorphological differences of the implant-abutment junction and their clinical implications: A pilot study. Clin. Oral Implants Res..

[35] Juodzbalys G., Sapragoniene M., Wennerberg A., Baltrukonis T. (2007). Titanium dental implant surface micromorphology optimization. J. Oral Implantol..

[36] Chung S.H., Heo S.J., Koak J.Y., Kim S.K., Lee J.B., Han J.S., Han J.H., Rhyu I.C., Lee S.J. (2008). Effects of implant geometry and surface treatment on osseointegration after functional loading: A dog study. J. Oral Rehabil..

[37] Motoyoshi M., Yano S., Tsuruoka T., Shimizu N. (2005). Biomechanical effect of abutment on stability of orthodontic mini-implant. A finite element analysis. Clin. Oral Implants Res..

[38] Kong L., Liu B.L., Hu K.J., Li D.H., Song Y.L., Ma P., Yang J. (2006). Optimized thread pitch design and stress analysis of the cylinder screwed dental implant. Hua Xi Kou Qiang Yi Xue Za Zhi.

[39] Hansson S., Werke M. (2003). The implant thread as a retention element in cortical bone: The effect of thread size and thread profile: A finite element study. J. Biomech..

[40] Rashad A., Sadr-Eshkevari P., Weuster M., Schmitz I., Prochnow N., Maurer P. (2013). Material attrition and bone micromorphology after conventional and ultrasonic implant site preparation. Clin. Oral Implants Res..

[41] Ono T., Hayashi M., Sasaki F., Nakashima T. (2020). RANKL biology: Bone metabolism, the immune system, and beyond. Inflamm. Regen..

[42] Jiang Y., Jia T., Gong W., Wooley P.H., Yang S.Y. (2013). Titanium particle-challenged osteoblasts promote osteoclastogenesis and osteolysis in a murine model of periprosthestic osteolysis. Acta Biomater..

[43] Córdova L.A., Trichet V., Escriou V., Rosset P., Amiaud J., Battaglia S., Charrier C., Berreur M., Brion R., Gouin F. (2015). Inhibition of osteolysis and increase of bone formation after local administration of siRNA-targeting RANK in a polyethylene particle-induced osteolysis model. Acta Biomater..

[44] Eroglu C.N., Ertugrul A.S., Eskitascioglu M., Eskitascioglu G. (2016). Changes in the surface of bone and acid-etched and sandblasted implants following implantation and removal. Eur. J. Dent..

[45] Deppe H., Wolff C., Bauer F., Ruthenberg R., Sculean A., Mucke T. (2018). Dental implant surfaces after insertion in bone: An in vitro study in four commercial implant systems. Clin. Oral Investig..

[46] Choi M.G., Koh H.S., Kluess D., O’Connor D., Mathur A., Truskey G.A., Rubin J., Zhou D.X.F., Sung K.-L.P. (2005). Effects of titanium particle size on osteoblast functions in vitro and in vivo. Proc. Natl. Acad. Sci. USA.

[47] Stratton-Powell A.A., Pasko K.M., Brockett C.L., Tipper J.L. (2016). The biologic response to polyetheretherketone (PEEK) wear particles in total joint replacement: A systematic review. Clin. Orthop. Relat. Res..

[48] Yang S.Y., Ren W., Park Y., Sieving A., Hsu S., Nasser S., Wooley P.H. (2002). Diverse cellular and apoptotic responses to variant shapes of UHMWPE particles in a murine model of inflammation. Biomaterials.

[49] Lohmann C.H., Dean D.D., Köster G., Casasola D., Buchhorn G.H., Fink U., Schwartz Z., Boyan B.D. (2002). Ceramic and PMMA particles differentially affect osteoblast phenotype. Biomaterials.

[50] Klinder A., Seyfarth A., Hansmann D., Bader R., Jonitz-Heincke A. (2018). Inflammatory response of human peripheral blood mononuclear cells and osteoblasts incubated with metallic and ceramic submicron particles. Front. Immunol..

[51] Elias C.N., Rocha F.A., Nascimento A.L., Cohelo P.G. (2012). Influence of implant shape, surface morphology, surgical technique and bone quality on the primary stability of dental implants. J. Mech. Behav. Biomed. Mater..

[52] Zhang L., Haddouti E.M., Welle K., Burger C., Wirtz D.C., Schildberg F.A., Kabir K. (2020). The effects of biomaterial implant wear debris on osteoblasts. Front. Cell Dev. Biol..

[53] Li J., Hastings G., Black J., Hastings G.W. (2016). Oxide bioceramics: Inert ceramic materials in medicine and dentistry. Handbook of Biomaterial Properties.

[54] Bertoldi C., Pradelli J.M., Consolo U., Zaffe D. (2005). Release of elements from retrieved maxillofacial plates and screws. J. Mater. Sci. Mater. Med..

[55] Pérez-Granados A.M., Vaquero M.P. (2002). Silicon, aluminium, arsenic and lithium: Essentiality and human health implications. J. Nutr. Health Aging.

[56] Rondeau V. (2002). A review of epidemiologic studies on aluminum and silica in relation to Alzheimer’s disease and associated disorders. Rev. Environ. Health.

